# Navigating the Challenges of Delayed Subdural Hemorrhage and COVID-19: A Case Report

**DOI:** 10.7759/cureus.54853

**Published:** 2024-02-25

**Authors:** Saleh S Al Qahtani, Dunya Alfaraj, Mohammed O Alzayer, Zainab Juma, Mohamed Abdulla, Husain Faraj, Abdulla Juma, Mohamed M Moussa

**Affiliations:** 1 Internal Medicine Department, Najran University Hospital, Najran, SAU; 2 Emergency Department, Imam Abdulrahman Bin Faisal University, Dammam, SAU; 3 Emergency Department, King Fahad University Hospital, Dammam, SAU

**Keywords:** posttraumatic subdural hemorrhage, brain injury, traumatic fall, covid-19, delayed onset

## Abstract

The delayed onset of posttraumatic subdural hemorrhage (SDH) represents non-specific clinical features, complicating the diagnostic process, especially in individuals predisposed due to pre-existing risk factors and comorbidities. This case report delineates the medical trajectory of a 61-year-old female patient who sustained a traumatic fall, initially displaying neither clinical nor radiological signs indicative of hemorrhage. However, three weeks post-injury, she developed altered mental status, cephalgia, and emesis. Diagnostic imaging unveiled a significant bilateral acute-on-chronic subdural hemorrhage exerting pronounced mass effect and leading to obliteration of the basal cisterns. Complicating her clinical picture was a concurrent SARS-CoV-2 infection and a medical history of hypertension. Emergent neurosurgical intervention was undertaken, encompassing the creation of bilateral burr holes for drainage and the placement of subdural drains. The patient was managed with the requisite medical therapies. Post-operatively, the patient regained consciousness and exhibited significant neurological improvement. Follow-up imaging demonstrated complete resolution of the subdural hemorrhage, and the patient achieved a full recovery of cognitive function. This case underscores the critical necessity for vigilant surveillance for delayed SDH in patients lacking initial radiographic findings and advocates for individualized therapeutic approaches in patients with concurrent pathologies. Prompt recognition, timely neurosurgical management, and care are pivotal to optimizing outcomes in delayed posttraumatic SDH cases.

## Introduction

Posttraumatic subdural hemorrhage (SDH) is an accumulation of blood beneath the outer covering of the brain (dura mater) following trauma. This condition is a significant clinical concern worldwide, presenting primarily in individuals who have suffered head injuries [1]. The prevalence of SDH in those with head trauma is noted, with common causes including falls, vehicular accidents, sports injuries, or any form of blunt force to the head. As individuals age, or in the case of infants, the risk of SDH increases due to brain shrinkage or insufficient neck muscle strength, respectively, making the veins between the skull and brain more susceptible to tearing even from minor injuries [1,2].

The symptoms of SDH may vary depending on its classification as acute, subacute, or chronic. Acute SDH symptoms are severe and immediate, developing within minutes to hours after injury, and include headache, nausea, vomiting, dizziness, and possibly loss of consciousness if untreated. Subacute and chronic SDHs manifest symptoms more gradually, potentially leading to misdiagnosis as the signs can be mistaken for other conditions like stroke, brain tumor, or dementia [2,3]. Diagnosis is typically achieved through a detailed clinical assessment and confirmed via imaging studies such as computed tomography (CT) scans or magnetic resonance imaging, which provide clear images of the brain to identify the location and extent of hemorrhage [4,5].

The management of SDH aims at stabilizing the patient while addressing the underlying cause of the hemorrhage. Immediate care involves managing the airway, breathing, and circulation. Surgical interventions, such as craniotomy or the drilling of burr holes for chronic cases, are employed to relieve pressure on the brain and remove the accumulation of blood [6]. Non-surgical management may be appropriate for smaller hematomas, with treatment including rest, medication, and close monitoring. The prognosis for SDH patients varies significantly based on the hematoma's size, the patient's age, and the promptness of treatment. While acute SDH carries a higher risk of mortality and long-term disability, chronic SDH often has a more favorable outcome, particularly if symptoms are mild and treatment is timely [3,7].

## Case presentation

**Figure 1:**
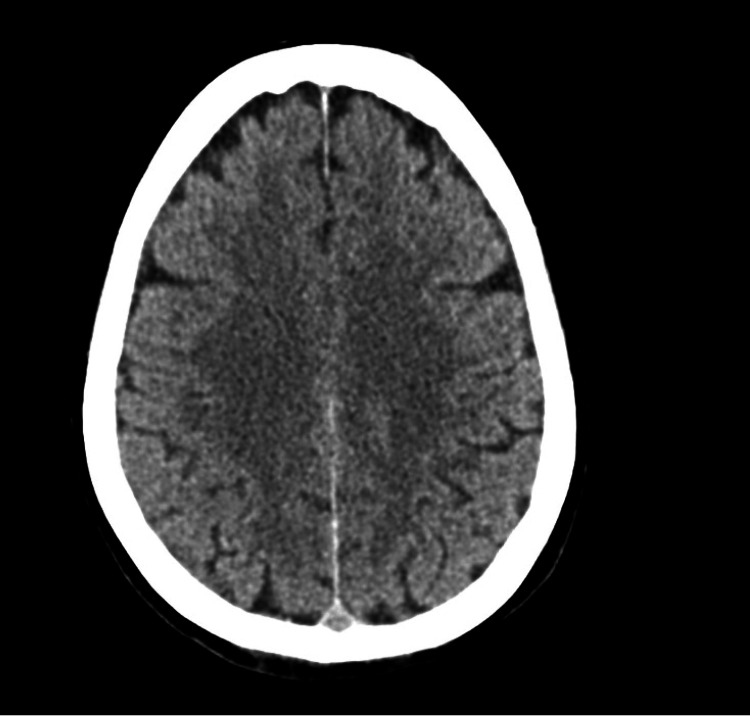
Initial computed tomography of the head showed no signs of any lesion

A 61-year-old woman with a medical history of controlled hypertension and diabetes mellitus experienced a fall, leading to her presentation at the emergency department. Initially, she was alert and conscious, and a non-enhanced CT scan of her brain showed no signs of trauma-related injury, resulting in her discharge (Figure 1). However, three weeks after falling, she returned with altered consciousness, headache, and vomiting. Notably, her medical background and family history were free of similar conditions.

**Figure 2:**
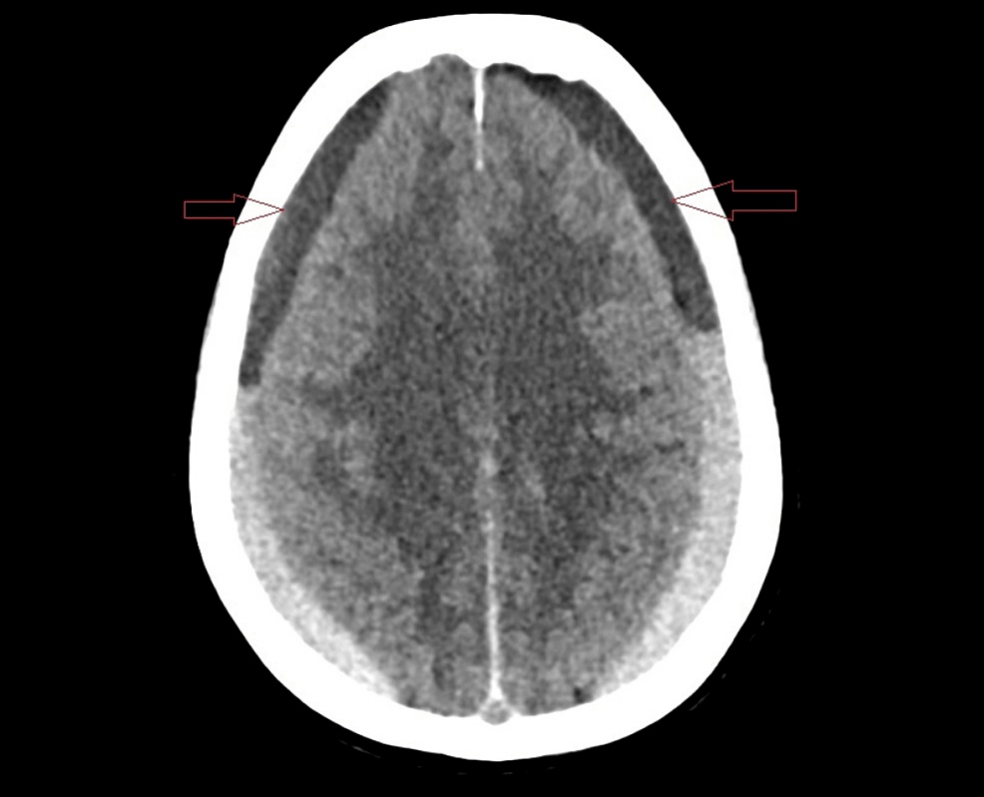
Computed tomography of the head during her second presentation showing signs of subdural hematoma (red arrows)

Upon her second presentation, the patient's condition had notably deteriorated, exhibiting drowsiness, hypertension, and a positive pronator drift test, indicative of neurological impairment. Subsequent imaging revealed an extensive bilateral acute-on-top-of-chronic subdural hemorrhage with significant complications, including mass effect, basal cistern obliteration, and herniation. An additional CT scan showed signs of subdural hematoma (Figure 2). This diagnostic phase underscored the critical role of follow-up evaluations and advanced imaging techniques in detecting delayed complications of trauma, especially in patients with pre-existing health conditions that may exacerbate their risk.

The treatment approach was aggressive and multidisciplinary including neurosurgery, internal medicine, radiology, anesthesia, and intensive care specialists, involving urgent neurosurgical intervention to relieve intracranial pressure and manage the hemorrhage, alongside medical management for her hypertension, diabetes, and newly diagnosed COVID-19 infection. Following surgery, the patient showed remarkable recovery, with follow-up imaging confirming the resolution of the hemorrhage. Her discharge and subsequent outpatient follow-ups highlighted the effectiveness of the treatment plan and the importance of patient education on preventive measures to avoid future incidents. This case exemplifies the complexities of managing delayed posttraumatic complications in patients with comorbidities, emphasizing the need for a comprehensive and tailored treatment strategy to ensure optimal outcomes.

## Discussion

SDH typically results from rapid clot formation beneath the inner layer of the dura mater yet external to the brain and arachnoid membrane [8]. A delayed acute SDH is characterized as an acute SDH not evident on initial CT scans but appearing on subsequent imaging. This phenomenon predominantly occurs in middle-aged and elderly individuals, often those on anticoagulant or antiplatelet therapy. Typically, these patients present with a Glasgow Coma Scale score of 15, showing no immediate posttraumatic loss of consciousness [1]. As demonstrated in our case, the patient initially exhibited no signs of hemorrhage with clear radiological imaging immediately after trauma. However, within three weeks, her condition declined, marked by altered consciousness, vomiting, nausea, and a positive pronator drift test.

Delayed traumatic subdural hemorrhages are identified as emerging high-density parenchymal lesions in previously normal or minimally abnormal regions, as described by Gudeman et al. [9] and further elaborated by Fukamachi et al. [10], emphasizing the critical role of imaging in diagnosing delayed SDH. In this case, initial CT scanning conducted immediately post-fall showed no traumatic brain injury. Yet, a follow-up CT scan three weeks later revealed extensive bilateral acute-on-chronic subdural hematomas, highlighting significant mass effects and complications like basal cistern obliteration and herniation.

Surgical intervention, particularly the drainage of chronic and subacute subdural hematomas via burr holes, has been associated with a low recurrence rate in long-term studies [11]. In the specific context of our patient, bilateral frontal/parietal burr holes were drilled for drainage, complemented by bilateral drain insertion to mitigate the recurrence risk. Moreover, the interrelation of COVID-19 with increased SDH risk, as suggested by studies such as that by Altschul et al., points to the potential vascular and autoregulatory disruptions caused by SARS-CoV-2, especially in patients with pre-existing conditions like hypertension [12]. This backdrop underscores the complexity of managing SDH in COVID-19-positive patients. Post-treatment, the patient showed complete hematoma resolution on follow-up CT scans, with no subsequent recurrence noted within three months, aligning with Lutz et al. findings on the timeline for hematoma recurrence [13]. Discharged with instructions on fall prevention and without the need for regular follow-up, the patient's case exemplifies the nuanced approach required in managing delayed acute SDH, particularly in the context of COVID-19.

Acute SDH typically presents with mixed hyper or hypodense areas on CT scans, reflecting unclotted blood or serum extruded during clot retraction. In some cases, especially with anticoagulation or coagulopathies, acute SDH may be nearly isodense with the adjacent cerebral cortex, making detection challenging. As the clot ages, the density of subdural hemorrhage starts to drop, and between 3 and 21 days, it becomes isodense to the cortex, necessitating contrast-enhanced CT or MRI for identification. Chronic SDH, defined as being at least three weeks old, becomes hypodense to the cortex and may mimic a subdural hygroma on imaging. In cases of acute on chronic SDH, a second episode of acute hemorrhage into a pre-existing chronic SDH typically appears as a hypodense collection with a hematocrit level located posteriorly. MRI further characterizes SDH with varying signal intensities depending on the age of the hematoma making it the most accurate in determining its characteristics [14].

The importance of meticulous follow-up evaluations and the utilization of advanced imaging techniques are pivotal in the management of patients with traumatic brain injuries, particularly in the context of delayed complications. Follow-up evaluations serve as a critical tool in monitoring the progression or resolution of injuries, allowing for the timely identification of delayed complications such as subdural hematomas. The role of serial imaging, utilizing modalities such as CT and MRI, is instrumental in detecting subtle changes that may not be apparent in the initial post-trauma period. These imaging techniques provide detailed visualization of brain structures, enabling the identification of evolving hematoma, brain swelling, or other secondary injuries that could influence patient management and prognosis [15].

Furthermore, the presence of pre-existing health conditions, such as anticoagulant therapy, hypertension, or diabetes, can significantly exacerbate the risk of delayed complications following trauma. These conditions may alter the natural healing process, increase the vulnerability to hemorrhagic events, or impact the cerebral vascular response to injury. Therefore, a comprehensive and individualized approach to patient care, which includes regular follow-up assessments and tailored imaging protocols, is essential to mitigate the risk of adverse outcomes in this patient population. The integration of clinical vigilance and advanced diagnostic tools is crucial in navigating the complexities of traumatic brain injuries and ensuring optimal patient outcomes [16].

## Conclusions

Delayed subdural hematoma (DSH) represents a complex clinical entity that, without timely diagnosis and intervention, may lead to adverse outcomes. The intricacies of managing DSH, especially when compounded by factors such as anticoagulant therapy and infectious diseases like COVID-19, necessitate a vigilant and comprehensive approach to care. This case exemplifies the potential for recovery with appropriate and prompt surgical and medical management, underscoring the critical need for healthcare professionals to maintain a high index of suspicion for DSH in patients presenting with relevant risk factors and clinical histories.
